# Efficacy of interleukin-6 in combination with D-dimer in predicting early poor postoperative prognosis after acute stanford type a aortic dissection

**DOI:** 10.1186/s13019-020-01206-y

**Published:** 2020-07-16

**Authors:** Qingsong Wu, Jiahui Li, Liangwan Chen, Liang Liang Yan, Zhihuang Qiu, Yue Shen, Xianbiao Xie, Linfeng Xie

**Affiliations:** 1 Department of Cardiac Surgery, Union Hospital, Fujian Medical University, Fuzhou, Fujian 350001 People’s Republic of China; 2 Fujian Medical University, Fuzhou, Fujian 350001 People’s Republic of China

**Keywords:** Acute Stanford a aortic dissection, Interleukin-6, D-dimer, Prognostic factor, Predictive value

## Abstract

**Background:**

We studied early poor postoperative prognosis in acute Stanford type A aortic dissection (ATAAD) patients and investigated the predictive effect of interleukin-6 (IL-6) combined with D-dimer in the early poor postoperative prognosis after ATAAD.

**Methods:**

Data on 141 ATAAD patients, who underwent emergency surgery between January 2018 and December 2018 at our hospital, were studied. We analyzed early postoperative prognosis using two patient groups. Patients with good prognosis were included in group A and those with poor prognosis were in group B. Univariate logistic and multivariable logistic regression analysis were performed for poor early postoperative prognosis.

**Results:**

Preoperative IL-6 level was lower (57.8 ± 39.0 vs 211.0 ± 153.7 pg/mL, *p* < 0.001) and the D-dimer was also lower (7.3 ± 6.1 vs. 16.7 ± 5.8 μg/mL, *p* < 0.001) in group A than in B. The cut-off points, determined by the ROC curve, were preoperative IL-6 > 108 pg/mL (area under the curve: AUC = 0.901) and D-dimer > 14.0 μg/mL (AUC = 0.817). Univariate logistic regression analysis showed that IL-6 > 108 pg/mL, D-dimer > 14.0 μg/mL, prothrombin time > 15 s, creatinine > 135 mmol/mL, and operation time > 306 min for ATAAD appeared to be early postoperative risk factors of poor prognosis. Multivariable logistic regression analysis showed that IL-6 > 108 pg/mL and D-dimer > 14.0 μg/mL were early postoperative risk factors for poor prognosis after ATAAD, and the odds ratios (ORs) of IL-6 > 108 pg/mL and D-dimer > 14.0 μg/mL were 24.937 (6.837, 90.931) and 18.757 (5.094, 69.075), respectively. When IL-6 was > 108 pg/mL (AUC = 0.901), the sensitivity and specificity of predicting early postoperative prognosis after ATAAD were 79.4 and 89.7%, respectively (95% confidence interval [CI] 0.839 to 0.963). When D-dimer was > 14.0 g/mL (AUC = 0.817), the sensitivity and specificity were 82.4 and 84.1%, respectively (95% CI 0.731 to 0.903). When combined with D-dimer (AUC = 0.936) (95% CI 0.793 to 0.979), the AUC values were more predictive than those for the individual marker.

**Conclusion:**

IL-6 > 108 pg/mL and D-dimer > 14.0 μg/mL is of high predictive value for the assessment of early poor postoperative prognosis after ATAAD. And IL-6 > 108 pg/mL in combination with D-dimer > 14.0 μg/mL is of higher predictive value.

## Introduction

Acute Stanford type A aortic dissection (ATAAD) is a serious cardiovascular disease with a dangerous time course, rapid progression, and high mortality. Emergency surgery is the only effective treatment. Despite the continuous improvement of surgical techniques, surgical complications and mortality rates remain high [1–3].

Presently, no sensitive preoperative predictors for ATAAD patients with postoperative outcome assessments need. There is an urgent need for a variety of joint clinical predictors of the prognosis of patients with ATAAD to improve perioperative treatment and to help assess early ATAAD postoperative prognosis better. In addition, clinical predictors will optimize the treatment strategies, improve the early surgical treatment of ATAAD, reduce serious early postoperative complications, and reduce the incidence of poor early postoperative prognosis. It is also helpful for more accurate and precise consultations with patients and families before surgery, so that the family of the patient can fully understand the critical condition of the patient effectively; it is good for reducing some medical disputes.

Coagulation and inflammatory response mechanisms are important in the pathogenesis and prognosis of aortic dissection. Relevant studies have reported the sensitivity of D-dimer to the diagnosis of acute aortic dissection, and the elevation of D-dimer also affects the prognosis of acute aortic dissection. However, as a single indicator, the accuracy of D-dimer is insufficient, and its systematic evaluation in combination with other indicators may be required [4–6]. A large number of basic studies have shown that interleukin-6 (IL-6) is related to the pathology of aortic dissection. There have also been reports in clinical studies regarding effect of elevated IL-6 on the occurrence of related complications after cardiovascular surgery [3, 7, 8].

The combination of IL-6 with D-dimer can provide valuable clinical predictive information for early poor postoperative prognosis after ATAAD and the combination has not been studied systematically. We therefore aimed to study the early poor postoperative prognosis in patients with ATAAD and to investigate the predictive effect of interleukin-6 (IL-6) combined with D-dimer in the early poor postoperative prognosis of ATAAD.

## Material and methods

### Study population

The preoperative IL-6 and D-dimer levels from 141 patients with emergency surgical treatment for ATAAD, from January to December 2018, were retrospectively collected. Postoperative complications were divided into two groups according to the Classification of Surgical Complications^9^ [9]: Those patients with good prognosis (Group A = Grade I, II, III) and those who had poor prognosis (group B = Grade IV, V) (Table 1).

**Table 1:** Classification of Surgical complications

Grade Defifinition	
Grade I Any deviation from the normal postoperative course without the need for pharmacological treatment or surgical, endoscopic, and radiological interventions Allowed therapeutic regimens are: drugs as antiemetics, antipyretics, analgetics, diuretics, electrolytes, and physiotherapy. This grade also includes wound infections opened at the bedside	
Grade II Requiring pharmacological treatment with drugs other than such allowed for grade I complications Blood transfusions and total parenteral nutrition are also included	
Grade III Requiring surgical, endoscopic or radiological intervention	
Grade IIIa Intervention not under general anesthesia	
Grade IIIb Intervention under general anesthesia	
Grade IV Life-threatening complication (including CNS complications)^a^ requiring IC/ICU management	
Grade IVa Single organ dysfunction (including dialysis)	
Grade IVb Multiorgan dysfunction	
Grade V Death of a patient	

*CNS* central nervous system, *IC* intermediate care, *ICU* intensive care unit

^a^Brain hemorrhage, ischemic stroke, subarrachnoidal bleeding, but excluding transient ischemic attacks

We included patients in which ATAAD was confirmed by CT angiography (CTA) or magnetic resonance angiography (MRA) and emergency treatment was performed with aortic root treatment. This includes aortic sinus formation, bentall procedure, AVR, CABG, David procedure or Carbol procedure + ascending aorta replacement + hemiarch replacement + a self-adaptive triple-branched stent graft implantation or ascending aorta replacement + hemiarch replacement + a self-adaptive triple-branched stent graft implantation. We excluded patients who had been hospitalized for more than 48 h after onset, those with incomplete data, and patients with a history of malignant tumors or chronic single-organ dysfunction. This study was approved by the ethics committee of our hospital and conformed to the Declaration of Helsinki.

### Definitions

A poor postoperative prognosis was defined a single organ dysfunction (including dialysis), multiorgan dysfunction (MODS) and death. MODS was defined as acute and potentially reversible dysfunction of two or more organ systems due to a variety of different clinical factors [10]. Malperfusion syndrome (MPS) was presented as sequela of localized or systemic ischemia (because of compromised blood flow to a visceral vessel or a limb) at admission [11, 12].

An emergency operation was defined as urgent condition by the doctor after the assessment of the need for surgery in the shortest time, or if there was a life in danger. Aortic root treatment included aortic sinus formation, bentall procedure, AVR, CABG, David procedure, and Carbol procedure.

### Serum measurements

All patients were admitted to the hospital (within 48 h after onset, without medication), and venous blood was extracted before surgery.

### Operative treatment [13, 14]

The details of the surgical procedure performed with aortic root treatment (includes aortic sinus formation, bentall procedure, AVR, CABG, David procedure orCarbol procedure) + ascending aorta replacement + Hemiarch Replacement + a self-adaptive triple-branched stent graft implantation have been described. All patients underwent total cardiopulmonary bypass.

The preoperative 25 indexes of all patients were observed and summarized. Preoperative indicators included age, gender, body mass index (BMI), IL-6, D- dimer, leukocyte, neutrophil granulocyte, lymphocyte, hemoglobin, albumin, prothrombin time (PT) and the NT-ProBNP, troponin I, serum creatinine, lactic acid, alanine aminotransferase, aspartate aminotransferase, preoperative ejection fraction (EF), Marfan syndrome, hypertension, diabetes, pericardial effusion (medium or above), organ perfusion, aortic regurgitation (AR) (moderate or above) and a history of cardiac surgery. Six intraoperative indicators were operative methods of the aortic root and operative data (operation, cardiopulmonary bypass (CPB), aortic clamp, selective cerebral perfusion (SBP),and circulatory arrest (CA) times) (Tables 2 and 3). Early postoperative prognosis, using the hierarchical classification based on Complications of Surgical Complications in two groups, was divided between Group A with good prognosis and Group B with poor prognosis.

**Table 2:** Preoperative data on the two patient group

Valuables	Group A (***n*** = 107)	Group B (***n*** = 34)	***P*** value
Male, n (%)	80 (74.8)	25 (73.5)	0.885
Age,n (year)	51.6 ± 10.6	50.6 ± 11.9	0.660
BMI (Kg/Mˆ2)	25.8 ± 3.9	25.6 ± 4.2	0.754
IL-6(pg/mL)	57.8 ± 39.0	211.0 ± 153.7	**<0.001**
D-dimer (ug/mL)	7.3 ± 6.1	16.7 ± 5.8	**<0.001**
Leukocyte(10ˆ9/L)	12.3 ± 4.0	12.8 ± 4.7	0.540
Neutrophile granulocyte(10ˆ9/L)	10.3 ± 3.9	10.8 ± 4.3	0.553
Leukomonocyte (10ˆ9/L)	1.1 ± 0.5	1.1 ± 0.6	0.924
PT (sec)	15.7 ± 13.7	15.7 ± 4.9	0.982
Heamoglobin(g/L)	129.5 ± 23.6	126.7 ± 23.3	0.553
Albumin(g/L)	37.3 ± 5.4	35.4 ± 4.6	0.056
Troponin-I (ug/L)	0.3 ± 1.0	1.9 ± 6.5	0.158
NT-ProBNP (pg/mL)	704.1 ± 1156.7	1993.0 ± 6129.2	0.231
Serum creatinine (umol/L)	93.1 ± 42.4	136.5 ± 127.7	0.060
Lactic acid (mmol/L)	2.0 ± 1.3	1.6 ± 1.3	0.178
Alanine aminotransferase (IU/L)	63.0 ± 169.8	166.5 ± 473.0	0.219
Aspartate aminotransferase (IU/L)	69.0 ± 226.2	190.4 ± 456.3	0.145
EF (%)	63.8 ± 7.0	62.7 ± 6.2	0.371
Marfan Syndrome, n (%)	5 (4.7)	2 (5.9%)	0.865
Hypertension, n (%)	79 (73.8)	30 (88.2)	0.080
Diabetes, n (%)	9 (8.4)	1 (2.9)	0.485
Pericardial effusion (Medium or above), n (%)	5 (4.7)	4 (11.8)	0.715
MPS, n (%)	7 (6.5)	4 (11.8)	0.534
AR (Medium or above), n (%)	28 (26.2)	7 (20.6)	0.512
History of cardiac surgery, n (%)	8 (7.5)	4 (11.8)	0.669

Continuous normally distributed variables were expressed as mean(±standard deviation) and not-normally distributed variables as medians (interquartile range)

**Table 3:** Surgical data on the two patient groups

Valuables	Group A (***n*** = 107)	Group B (***n*** = 34)	***P*** value
Intraoperative time			
Operative time (min)	302.2 ± 61.8	317.1 ± 57.8	0.205
CPB time (min)	148.6 ± 38	149.8 ± 38.1	0.874
Aortic Clamp time (min)	49.2 ± 18.9	55.7 ± 25.9	0.187
SBP time (min)	9.6 ± 3.2	10.1 ± 4.3	0.474
CA time (min)	2.8 ± 0.8	3.2 ± 1.3	0.155
Aortic Root Concomitant procedure			
Bentall,n(%)	24 (22.4)	6 (17.6)	0.724
AVR,n (%)	1 (0.9)	0 (0.0)	0.544
Cabrol,n(%)	0 (0.0)	1 (2.9)	0.544
CABG,n(%)	3 (2.8)	4 (11.8)	0.100
David,n(%)	1 (0.9)	0 (0.0)	0.544
Sinus forming,n (%)	47 (43.9)	10 (29.4)	0.193

Continuous normally distributed variables were expressed as mean (±standard deviation) and not-normally distributed variables as medians (interquartile range)

Continuous variables were analyzed using Mann-Whitney U test and presented by the mean + standard deviation, or mean (min, max), while categorical variables were tested by a chi-squared test or Fisher’s exact test. According to receiver operating characteristics (ROC) curve, cut-off point was selected, and variables with a *p*-value < 0.10 for a single factor were selected for logistic regression analysis of related risk factors in patients. *P*-values < 0.05 were considered statistically significant. All data were statistically processed using the SPSS 19.0 statistical software package.

## Results

In 141 patients with ATAAD cases, poor early postoperative prognosis was seen in 34 (24.1%). Of these, renal failure involved 17 (12.1%) patients, respiratory failure, 3 (2.1%), liver failure, 1 (1.4%), digestive system dysfunction in 1 (0.7%), nervous system dysfunction (0.7%), sepsis, 1 (0.7%), and MODS 2 (1.4%) cases. While 8 (5.7%) patients died, there were 107 cases with good postoperative prognosis.

**Fig. 1:**
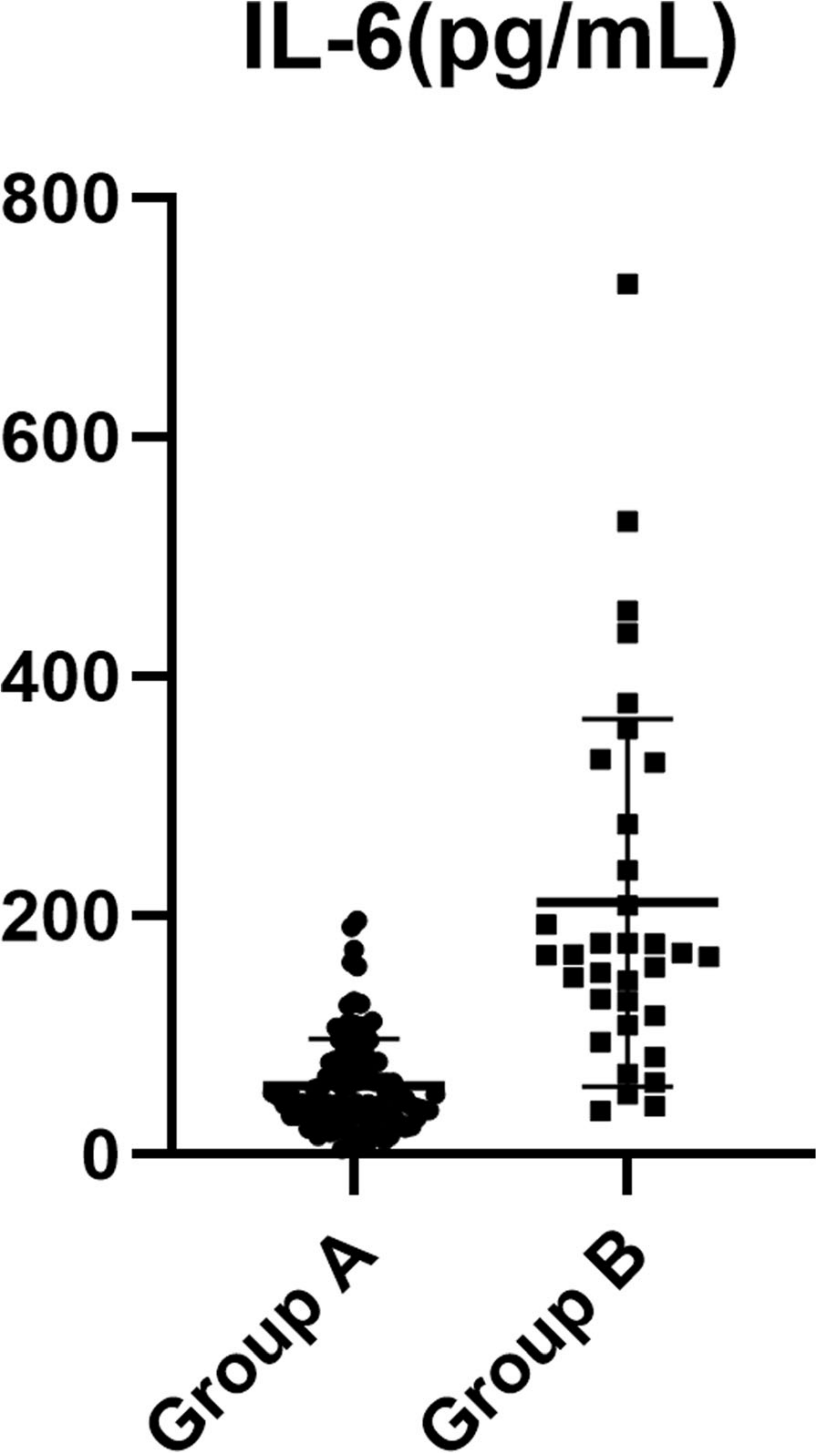
Distribution of interleukin-6 levels in A and B groups

**Fig. 2:**
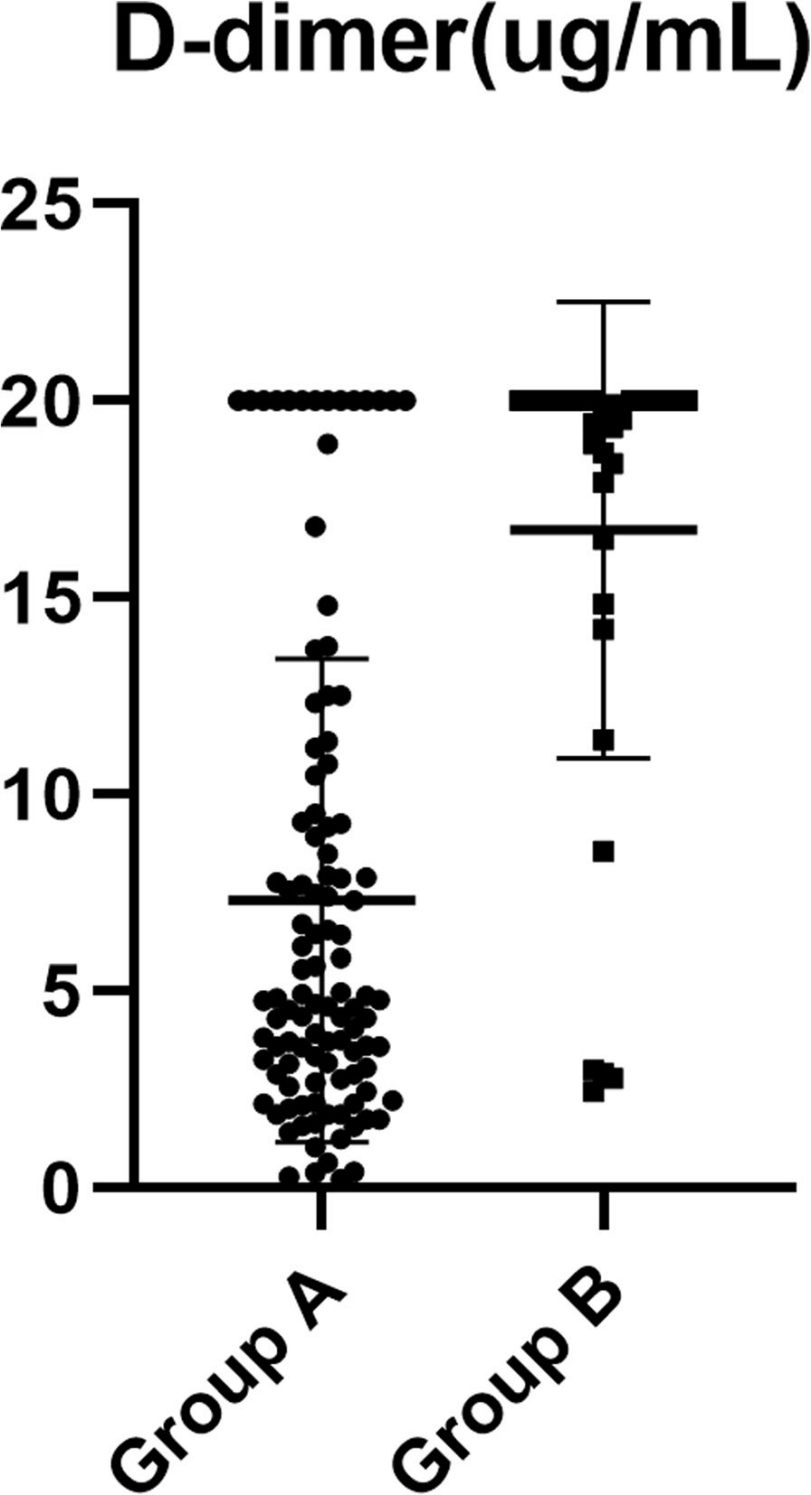
Distribution of D-dimer levels in A and B groups

**Fig. 3:**
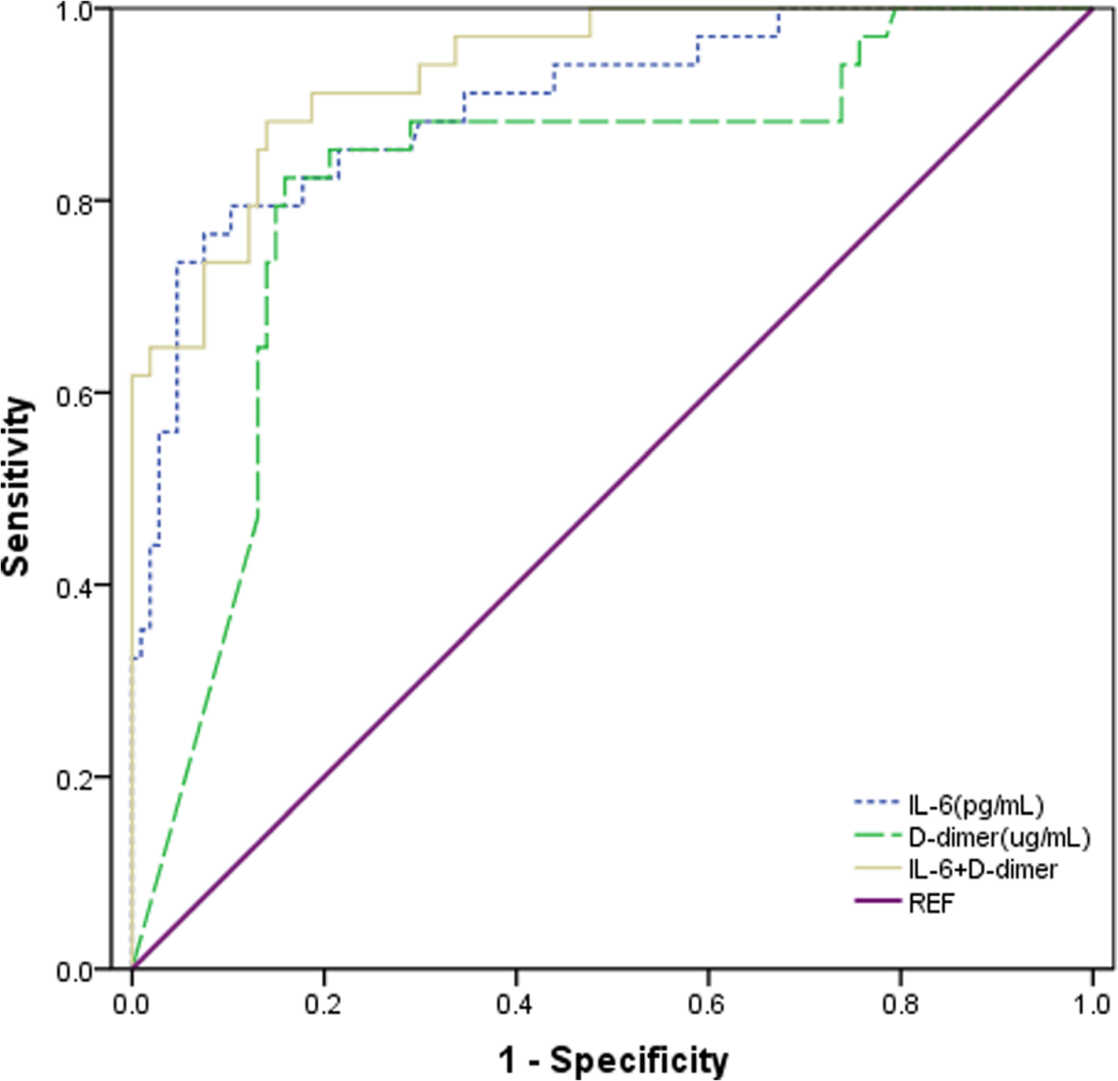
Receiver-operating characteristic curve in the prediction of the independent risk factors of early poor postoperative prognosis after Acute Stanford type A aortic dissection

Preoperative IL-6 level was lower (57.8 ± 39.0 vs. 211.0 ± 153.7 pg/mL, *P* < 0.001) and D-dimer level was also lower (7.3 ± 6.1 vs. 16.7 ± 5.8 μg/mL, *P* < 0.001) in group A than in group B. The cut-off points, determined by ROC curve, where preoperative IL-6 was > 108 pg/mL (area under the curve: AUC = 0.901) and D-dimer > 14.0 μg/mL (AUC = 0.817) (Tables 2 and 5, Figs. 1, 2, and 3).

**Fig. 4:**
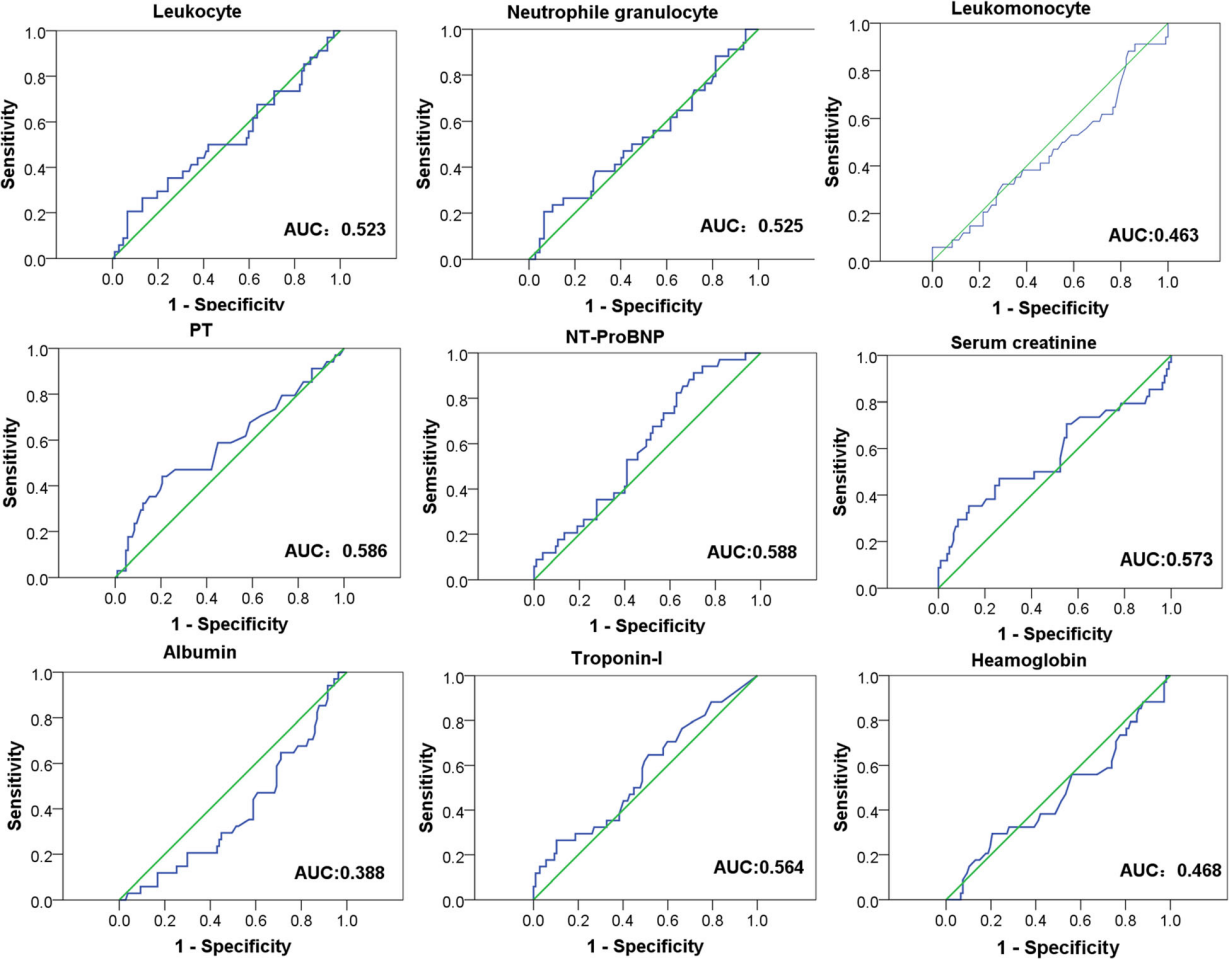
Receiver-operating characteristic curve

Univariate analysis of poor prognosis showed that IL-6 > 108 pg/mL, D-dimer > 14.0 μg/mL, PT > 15 s, creatinine tend for greater than 135 mL and operation time > 306 min were predictors of adverse postoperative prognostic risk factors (Table 3 and Fig. 4). Multiple factor analysis showed that IL-6 > 108 pg/mL, D-dimer > 14.0 μg/mL were the independent risk factors of early poor postoperative prognosis after ATAAD (Table 4). ORs of IL-6 > 108 pg/mL and D-dimer > 14.0 μg/mL were 24.937 (6.837, 90.931) and 18.757 (5.094, 69.075), respectively. The sensitivity and specificity of IL-6 > 108 pg/mL for early adverse prognosis after ATAAD were 91.2 and 89.7%, respectively (95% CI 0.839 to 0.963). The sensitivity and specificity of D-dimer > 14.0 μg/mL were 88.2 and 82.2%, respectively (95% CI 0.731 to 0.903) (Tables 4 and 5, Fig. 3).

**Table 4:** Univariate and multivariate analyses for all patients who underwent surgery

Variable	Univariate Model			Multivariate Model		
	OR	95% CI	***p-value***	OR	95% CI	***p-value***
**Age, years**						
< 60	REF					
≥ 60	1.016	0.423–2.438	0.885			
**BMI, kg/m**						
<25	REF					
≥ 25	0.621	0.286–1.349	0.228			
**Sex**						
Female	REF					
Male	1.067	0.443–2.566	0.885			
**IL-6(pg/mL)**						
≤ 108	REF			REF		
>108	33.662	11.906–95.178	**<0.001**	24.937	6.837–90.931	**<0.001**
**D-dimer (ug/mL)**						
≤ 14.0	REF			REF		
>14.0	24.706	8.884–68.705	**<0.001**	18.757	5.094–69.075	**<0.001**
**PT (sec)**						
≤ 15.0	REF			REF		
> 15.0	2.667	1.12–6.346	**0.027**	1.198	0.268–5.350	0.813
**Heamoglobin (10ˆ9/L)**						
≤ 10	REF					
>10	0.979	0.419–2.285	0.961			
**Neutrophile granulocyte (10ˆ9/L)**						
≤ 7.0	REF					
>7.0	0.942	0.361–2.456	0.942			
**Leukomonocyte (10ˆ9/L)**						
≤ 0.8	REF					
>0.8	0.545	0.241–1.235	0.146			
**Hemoglobin (g/L)**						
≥ 120	REF					
<120	0.77	0.326–1.820	0.552			
**Albumin (g/L)**						
≥ 35.0	REF					
<35.0	0.565	0.256–1.427	0.157			
**Troponin-I (ug/L)**						
≤ 0.02	REF					
>0.02	1.229	0.568–2.662	0.601			
**NT-ProBNP (pg/mL)**						
≤ 349	REF					
>349	1.442	0.663–3.135	0.356			
**Serum creatinine (umol/L)**						
≤ 135	REF			REF		
>135	**2.365**	**0.982–5.696**	**0.055**	1.965	0.493–7.829	0.338
Lactic acid (mmol/L)						
≤ 2.5	REF					
>2.5	2.287	0.735–7.116	0.153			
Alanine aminotransferase (IU/L)						
≤ 40	REF					
>40	0.650	0.285–1.483	0.306			
Aspartate aminotransferase (IU/L)						
≤ 46	REF					
>46	0.605	0.258–1.415	0.246			
**EF (%)**						
≥ 60.0	REF					
<60.0	1.128	0.431–2.954	0.807			
**Marfan Syndrome**						
no	REF					
yes	1.275	0.236–6.891	0.778			
**Hypertension**						
no	REF					
yes	2.469	0.505–12.080	0.265			
**Diabetes**						
no	REF					
yes	0.33	0.040–2.703	0.302			
**Pericardial effusion (Medium or above)**						
no	REF					
yes	3.281	0.444–24.234	0.244			
**MPS**						
no	REF					
yes	1.877	0.583–6.043	0.291			
**AR (Medium or above)**						
no	REF					
yes	0.731	0.287–1.866				
**History of cardiac surgery**						
no	REF					
yes	1.65	0.464–5.863	0.439			
**Operative time (min)**						
≤ 306	REF			REF		
>306	**1.962**	**0.899–4.279**	**0.09**	3.635	0.977–13.521	0.054
**CPB time (min)**						
≤ 149	REF					
>149	0.894	0.409–1.952	0.779			
**Aortic Clamp time (min)**						
≤ 51	REF					
>51	1.324	0.601–2.920	0.486			
**SBP time (min)**						
≤ 9.7	REF					
>9.7	1.037	0.468–2.296	0.929			
**CA time (min)**						
≤ 3.1	REF					
>3.1	1.121	0.489–2.568	0.787			
**Aortic Root Concomitant procedure**						
no	REF					
yes	0.659	0.294–1.478	0.312

**Table 5:** Diagnostic value of IL-6 and D-dimer for poor postoperative prognosis

	AUC	Cut-off value	95% CI	***P*** value
IL-6 (pg/mL)	0.901	108.0	0.839 to 0.963	<0.001
D-dimer (ug/mL)	0.817	14.0	0.731 to 0.903	<0.001
IL-6 + D-dimer	0.936		0.793 to 0.979	<0.001

When IL-6 was combined with D-dimer, the AUC reached 0.936 (95% CI 0.793 to 0.979). The AUC values for the combined indicators were more predictive than with the individual marker alone (Fig. 3).

In summary, IL-6 and D-dimer were strong predictors of early poor postoperative prognosis after ATAAD, and they were more accurate when used in combination.

## Discussion

ATAAD is a fatal cardiovascular disease and poses a serious threat to human life and health [15]. Surgery is the treatment of choice, and without surgery, the mortality rate of ATAAD within 48 h is 50%. However, the mortality rate remains high after surgery due to many postoperative complications [16]. Relevant studies have shown that most patients with ATAAD had increased IL-6 before surgery. Intercellular IL-6 is an inflammatory cytokine, which plays an important role in inflammation and immune response and is closely related to cardiovascular diseases [17]. A functional multipotent prototype cytokine also has an important role in host defense. When infection or tissue damage occurs, IL-6 is rapidly produced by monocytes and macrophages; and it helps remove infectious agents and restore damaged tissues by activating immunity, blood, and acute phase reactions [18]. It has been found that there is an elevation in the early stage of acute aortic dissection, but the predictive value of the clinical outcome of acute aortic dissection is still lacking [19]. D-dimer is a cross-linked fibrin degradation product, suggesting that aortic dissection has fibrinolytic activity, which can be detected in peripheral blood within 10 min after the onset of disease. Various studies have confirmed the potential sensitivity and specificity of d-dimer, which is valuable in early diagnosis, differential diagnosis, and prognosis prediction [4, 5, 20–22]. However, few studies examined if D-dimer can provide a valuable clinical prediction for early complications after ATAAD, and evidence is needed to assess the combination of indicators for predicting risk. This study showed that preoperative IL-6 and D-dimer appear to predict early poor postoperative prognosis after ATAAD, and they have great prospective application in preoperative biochemical joint assessment. They can be very good for predicting poor early postoperative prognosis and have advantages of being rapid, non-invasive and easy to operate [23].

Preoperative concentrations of IL-6 and D-dimer in patients with ATAAD were measured, and it was found that there was statistical significance in indicators between the two groups A and B, which had clinical value in predicting early poor postoperative prognosis. IL-6 levels in groups A and group B was 57.8 ± 39.0 and 211.0 ± 153.7 pg/mL (*p* < 0.001), respectively, and corresponding D-dimer levels were 7.3 ± 6.1 and 16.7 ± 5.8 μg/mL (*p* < 0.001). ROC curve analysis showed that the truncation value of IL-6 was 108 pg/mL and that of D-dimer was 14.0 μg/mL. Single factor and multi-factor analysis revealed the ORs of plasma IL-6 > 108 pg/mL and D-dimer > 14.0 μg/mL (24.937 (6.837, 90.931);18.757 (5.094, 69.075) was a risk factor for poor postoperative prognosis after ATAAD. This result is helpful for more accurate and precise consultations with patients and families before surgery, so that the family of the patient can fully understand the critical condition of the patient effectively; it is good for reducing some medical disputes.

Most clinical symptoms in patients with ATAAD include sudden chest back pain, but, due to anatomical factors, a portion of patients show paroxysmal abdominal pain, waist pain, headache, dizziness, vomiting, hemiplegia, both lower limbs weakness, and hematochezia. These not only increase the difficulty of diagnosis, but also make it easy to misdiagnose and not diagnose [4, 24, 25]. ROC curve analysis also showed that the cut-off value of IL-6 was 108 pg/mL, the cut-off value of D-dimer was 14.0 μg/mL, and the AUC of IL-6 and D-dimer were 0.905 and 0.817, respectively. When IL-6 and D-dimer were used in combination, the AUC reached 0.936. The sensitivity and specificity of IL-6 > 108 pg/mL for early adverse prognosis after acute aortic dissection were 91.2 and 89.7%, respectively (95% CI 0.839 to 0.963). The sensitivity and specificity of D-dimer > 14.0 μg/mL were 88.2 and 82.2%, respectively (95% CI 0.731 to 0.903). (Table 4 and Fig. 3). In summary, IL-6 > 108 pg/mL and D-dimer > 14.0 μg/mL is of high predictive value for the assessment of early poor postoperative prognosis after ATAAD. And IL-6 > 108 pg/mL in combination with D-dimer > 14.0 μg/mL is of higher predictive value.

In addition, eight patients died in research group B, with a mortality rate of 5.67%. Single organ/multiple organ dysfunction included 28 cases (19.9%), while some patients have been accompanied by complications before operated such as poor perfusion syndrome (11.8%). However, our research shows that variation between patients with preoperative complications and intraoperative data of poor postoperative prognosis had no statistical significance (Tables 2 and 3). This is related to the improvement and technical level of our center in the surgical treatment of ATAAD. Relevant surgical procedures have been noted in the literature [13, 26]. However, we believe that future prospective cohort studies on preoperative assessment system of patients may help us in our hospital with the assessment for high-risk patients, we can be certain about decisions for perioperative interventional therapy. We can also perform the ascending aorta replacement + hemiarch replacement, give up total arch replacement, and include a self-adaptive triple-branched stent graft implantation. When the surgical operation time is short, there will be no need to be under deep low temperature circulatory arrest. It can save the patient’s life, this is also our plan in the future, creating more opportunities to help patients with wider range of replacement of aortic dissection, reduce the incidence of serious postoperative complications, and postoperative mortality.

### Limitations

In this study, a small sample was retrospectively analyzed, and its outcome could limit the statistics, but still has relevant statistical value. We hope to continue the preoperative IL-6/D-dimer combination for further research, including an increased sample size and more joint biochemical indexes to provide us with more accurate prediction of poor prognosis after ATAAD.

## Conclusions

IL-6 > 108 pg/mL and D-dimer > 14.0 μg/mL have high predictive value for the assessment of early poor postoperative prognosis after ATAAD. Moreover, IL-6 > 108 pg/mL in combination with D-dimer > 14.0 μg/mL is of higher predictive value.

## Data Availability

Please contact author for data requests.

## Abbreviations

ATAAD: Acute Stanford type A Aortic Dissection
IL-6: Interleukin-6
ROC: Receiver Operating Characteristic
ORs: Odds Ratios
AUC: Area under the curve
CTA: Computed Tomography angiography
MRA: Magnetic resonance angiography
CABG: Coronary Artery Bypass Grafting
AVR: Aortic valve placement
MODS: Multiorgan dysfunction
MPS: Malperfusion syndrome
CNS: Central nervous system
IC: Intermediate care
ICU: Intensive care unit
BMI: Body mass index
PT: Prothrombin time
EF: Left ventricular ejection fraction
NT-ProBNP: N-termininal forebrain natriuretic peptide
AR: Aortic regurgitation
CPB: Cardiopulmonary bypass time
SBP: Selective cerebral perfusion
CA: Circulatory Arrest
